# Neuro‐Behçet's Disease and Psychiatric Disorders: From a Case Report to a Systematic Review

**DOI:** 10.1002/brb3.71593

**Published:** 2026-07-23

**Authors:** Jorge Renau, Iván Echeverria, Ana Benito, Elena Sesé, María Vicenta Lucas‐Miralles, Marc Peraire

**Affiliations:** ^1^ TXP Research Group Universidad Cardenal Herrera‐CEU, CEU Universities Castellón de la Plana Spain; ^2^ Department of Mental Health Consorcio Hospitalario Provincial De Castellón Castellón de la Plana Spain; ^3^ Torrent Mental Health Unit Valencia University General Hospital Valencia Spain; ^4^ Pre‐Departmental Medicine Unit Universitat Jaume I Castellón de la Plana Spain

**Keywords:** Behçet's disease, case report, depression, mania, neuro‐Behçet's disease, psychiatric symptoms, psychosis, systematic review

## Abstract

**Background:**

Behçet's disease is a chronic, relapsing systemic vasculitis that can affect multiple organ systems. Neurological involvement, known as neuro‐Behçet's disease, occurs in a subset of patients, while psychiatric manifestations—termed neuro‐psycho Behçet's disease—remain poorly characterized. Understanding the clinical course, pathophysiology, and management of this condition is crucial for interdisciplinary care.

**Objective:**

To present a detailed case of a patient with neuro‐psycho Behçet's disease and to explore its pathophysiology, clinical features, psychiatric symptoms, temporal progression, and therapeutic considerations.

**Methods:**

A clinical case of a 42‐year‐old male with neuro‐Behçet's disease who developed a manic episode is described. Concurrently, a systematic review of published cases of neuro‐psycho Behçet's disease was conducted using the Web of Science, PubMed, and Embase databases on July 19, 2024, following the criteria of the PRISMA‐ScR Statement.

**Results:**

The clinical case illustrates a multiphasic disease course, with systemic Behçet's disease symptoms preceding neurological and subsequently psychiatric manifestations. The systematic review included thirty‐four cases, showing diverse psychiatric symptoms, including psychotic, affective, and behavioral disturbances. Neuroimaging frequently revealed parenchymal lesions correlating with psychiatric symptoms. Management typically combined immunosuppressive therapy for Behçet's disease with psychiatric treatment, including antipsychotic medications and mood stabilizers.

**Conclusions:**

Neuro‐psycho Behçet's disease represents a clinically and pathophysiologically complex entity. The correlation between parenchymal lesions and psychiatric symptoms supports the hypothesis of a shared neurobiological substrate mediated by inflammatory and immunological mechanisms. A biphasic model, in which initial vascular involvement evolves into neurochemical and structural dysfunction, provides a coherent framework for understanding the relationship between Behçet's disease and psychiatric symptoms.

## Graphical abstract

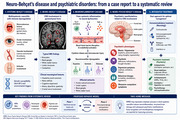

## 1 Introduction

Behçet's disease (BD) is a primary, chronic, relapsing systemic vasculitis that affects large and small vessels of the venous and arterial systems. The most characteristic symptom is recurrent orogenital ulcers, which may be accompanied by eye, skin, gastrointestinal, or joint lesions (Borhani‐Haghighi et al. 2020; Rodríguez‐Carrio et al. 2021). The disease is more common in men and typically begins around the second or third decade of life. Its aetiology is multifactorial, involving both genetic and environmental factors. In fact, in endemic areas, its presence is strongly correlated with the prevalence of human leukocyte antigen (HLA)‐B51. This makes its prevalence higher in East Asia, the Middle East, and the Mediterranean basin, hence its classical name, the ‘Silk Road disease’ (Borhani‐Haghighi et al. 2005; Talarico et al. 2015).

Neurological involvement occurs in approximately 9% of patients with BD (ranging from 3% to 30%) and predominantly affects the central nervous system (CNS). This variant, called neuro‐Behçet's disease (NBD), is more common in young men and tends to develop after the onset of systemic symptoms (Borhani‐Haghighi et al. 2020; Zhang et al. 2021). The aetiopathogenesis of NBD has been widely debated, although it is most likely a vasculitis that primarily, but not exclusively, affects the venules of the mesodiencephalic junction, cerebellar peduncles, and basal ganglia. Thus, inflammation resulting from perivascular infiltration of polymorphonuclear cells, mononuclear cells, T helper 17 cells (Th17), interleukin 6 (IL‐6), and tumor necrosis factor alpha (TNF‐α) would favor the destruction of vessels and neuronal apoptosis, leading to replacement with gliosis (Borhani‐Haghighi et al. 2020; Rodríguez‐Carrio et al. 2021). This aetiopathogenesis allows the symptoms of NBD to be divided into a parenchymal subtype and a non‐parenchymal or vascular subtype (Zhang et al. 2021).

The parenchymal subtype is the most common and mainly affects the brainstem (mesodiencephalic junction, followed by pontine‐bulbar), diencephalon, and, less frequently, periventricular and subcortical white matter (Saip et al. 2014). This gives rise to brainstem, hemispheric, spinal cord, and meningoencephalitic syndromes that present with a wide variety of neurological symptoms, such as headache (most common), sensorimotor indications, epileptic seizures, and dementia, among others. Parenchymal involvement can occur as an acute form, which is associated with a good response to corticosteroids, or as a chronic progressive form, which requires treatment with methotrexate. The chronic form typically presents with a persistent increase in IL‐6 levels in the cerebrospinal fluid and brainstem atrophy on magnetic resonance imaging (MRI) (Talarico et al. 2015).

Furthermore, parenchymal involvement has been associated with the occurrence of psychiatric symptoms in up to 2% of cases, including anxiety, affective disorders, and psychotic disorders (Akman‐Demir et al. 1999; Kirbaş et al. 2017). Although psychiatric manifestations are primarily associated with parenchymal involvement and the course of BD, the onset of psychiatric symptoms does not always coincide with acute systemic episodes. Instead, they may evolve relatively independently, suggesting either the presence of a continuously active pathological process within the CNS or the involvement of multiple etiopathogenic mechanisms in the context of a broader disease spectrum (Akman‐Demir et al. 1999; Bozikas et al. 2015; Kirbaş et al. 2017).

In this context, the term “neuro‐psycho Behçet's disease” (NPBD) (Kırbaş et al. 1988) was proposed as a descriptive construct to encompass patients with BD and associated psychiatric symptoms. However, NPBD concept does not constitute a formal nosological entity. Within this conceptual framework, NPBD shares similarities with other autoimmune disorders with neuropsychiatric involvement, such as anti‐NMDA receptor encephalitis, systemic lupus erythematosus, and multiple sclerosis (MS).

Despite the severity and relevance of NPBD, its etiopathogenic mechanisms, different presentations, and best therapeutic approaches to its treatment remain unknown. The first description of BD with psychiatric involvement occurred more than 50 years ago (Epstein et al. 1970), but no research has been conducted to systematically study this field, leaving gaps in our knowledge. Therefore, this paper presents the case of a male patient with NBD who presented a manic episode. We also conducted a systematic review of NPBD clinical cases published in the academic literature to highlight the relationship between NBD and psychiatric disorders, examining the mechanisms involved from a neuropsychiatric perspective.

## 2 Materials and Methods

This clinical case was selected and described by the same mental health professionals who treated the patient. For this purpose, the patient's consent to use data from their medical records was obtained.

### 2.1 Protocol and Registration

A systematic review was conducted following the PRISMA‐ScR Statement criteria and was registered in the PROSPERO database (CRD42024563024).

### 2.2 Search Strategy and Information Sources

The systematic review was completed on July 19, 2024, in the Web of Science, PubMed, and Embase databases, which collectively recover almost 96% of the relevant studies available (Bramer et al. 2017).

The search strategy used was (neurobehcet OR behcet) AND (psychos* OR hallucination OR delusion OR schizo* OR depress* OR mania* OR bipolar OR anxi* OR obsess*). The Derwent Innovations Index and Grants Index databases were excluded from the Web of Science search due to their lack of relevance to the field.

To ensure maximum sensitivity, MeSH/Emtree terms were not used. However, the selected databases interpret free‐text terms as their MeSH/Emtree equivalents via the automatic term mapping feature. No filters were added. Gray literature was not screened.

### 2.3 Eligibility Criteria

The PICOS (participants, intervention, context, outcomes, and study design) framework was used to establish eligibility criteria. We included (1) case reports or case series—regardless of whether they were published as articles, posters, or oral communications; (2) patients diagnosed with NBD who presented psychiatric disorders or symptoms; (3) articles written in English, Spanish, or any language that could be easily translated with an online translator. We excluded (1) cases in which the psychopathology could not be clearly attributed to NBD; (2) patients with a previous history of psychiatric disorders; (3) articles that provided the prevalence but did not describe the course of the disease or the treatment in detail; (4) articles whose language was not English or Spanish or could not be translated with an online translator.

### 2.4 Study Selection

**FIGURE 1:**
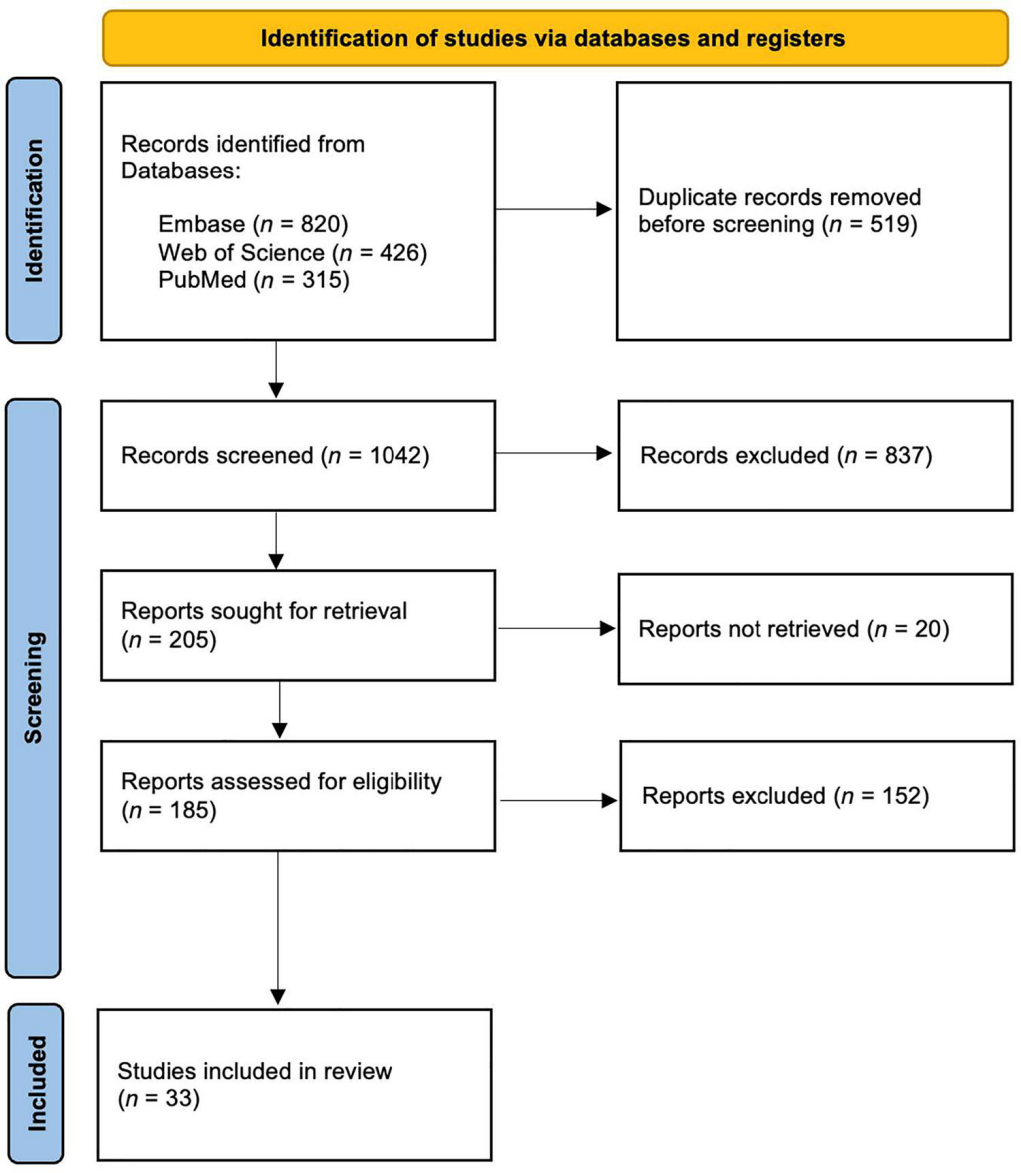
PRISMA flow diagram.

The references obtained from the search were entered into Rayyan systematic review management software, which identified duplicate studies that were then screened and discarded by one of the authors (I. E.). After removing the duplicates, two of the authors (J. R. and I. E.) independently screened the references. First, they reviewed the titles and abstracts, excluding any that did not meet the inclusion criteria or were not available. Then they evaluated the full‐text articles, excluding any not meeting the inclusion criteria or that were unavailable. Any resulting discrepancies were resolved by a third reviewer (M. P.) (Figure 1).

### 2.5 Data Extraction

Two authors (J.R. and I.E.) extracted data from the articles using a template: (1) study characteristics (author, country, and publication year); (2) patient sociodemographic data (age, sex, nationality, and medical history); (3) history of BD (medical signs and symptoms and relevant analytical tests); neuropsychiatric symptoms (presence and duration); imaging tests; management and treatment. Any resulting discrepancies were resolved by a third reviewer (M. P.).

### 2.6 Quality of Studies

As in previous studies (Bastos et al. 2021; Smith et al. 2021), two authors (J. R. and I. E.) independently assessed the case reports using the framework developed by Murad et al. (2018) to evaluate their methodological quality. This tool includes several domains (selection, ascertainment, causality, and reporting) with several criteria in each section. According to the methodological criteria met by the included cases, they were classified as low quality (between 0 and 2), medium quality (between 3 and 5), or high quality (more than 6).

### 2.7 Sensitivity Analysis

A qualitative sensitivity analysis was carried out, excluding low‐quality studies, to check whether the results found were maintained.

## 3 Results

### 3.1 Clinical Case

A 42‐year‐old Moroccan man, married with two children and no relevant family history, initially presented in June 2021 with recurrent oral ulcers.

In October 2022, he developed headache, epigastric pain, and a tonic–clonic seizure with aphasia, diplopia, and tetraparesis, requiring intensive care unit admission. EEG showed right temporal focal activity. MRI revealed bilateral temporal cortico‐subcortical edema and a bulbopontine junction lesion, along with signal alterations at C3 and C6–C7. CSF analysis demonstrated pleocytosis (1405/µL) and elevated protein (90 mg/dL). Acute disseminated encephalomyelitis (ADEM) was initially suspected, and he received ceftriaxone, acyclovir, and high‐dose corticosteroids, with partial improvement.

In May 2023, he presented with headache, diplopia, and right hemiparesis. MRI showed a pontine lesion with incomplete ring enhancement and bilateral temporal edema. Treatment with methylprednisolone and mycophenolate mofetil led to progressive recovery, although fatigue, hypoesthesia, and mild cognitive impairment (MMSE 23/30) persisted. Following admission to neurology, prednisone 50 mg (reduced by 10 mg every 15 days); mycophenolate mofetil, 500 mg every 12 h, and trimethoprim‐sulfamethoxazole 400/80 mg every 48 h; for 1 month, were prescribed.

Residual right‐sided hypoesthesia, mild dysmetria, hemiparetic gait, diplopia, hyperreflexia, and memory problems persisted over subsequent months. In November 2023, he presented a new relapse with right facio‐brachio‐crural hemiparesis and visual loss. CSF again showed elevated protein (107 mg/dL) and pleocytosis (1093 leukocytes/µL), and MRI demonstrated new hyperintense lesions in the pons and basal ganglia. Given the combination of recurrent oral ulcers, neurological involvement, and systemic inflammatory manifestations, a diagnosis of BD was established according to the International Criteria for Behçet's Disease (ICBD). Although the presentation was atypical (due to HLA‐B51 negativity), treatment with rituximab, mycophenolate, and prednisone was initiated.

Differential diagnosis encompassed ADEM, an inflammatory demyelinating disease such as MS, and parenchymal NBD. ADEM was initially favored but became unlikely due to recurrent relapses (versus its typical monophasic course), while MS was considered improbable given the brainstem/basal ganglia‐predominant lesion pattern, the absence of oligoclonal bands in the CSF, and the fulfillment of systemic Behçet's criteria. The subsequent clinical evolution—characterized by recurrent inflammatory relapses, systemic manifestations meeting Behçet's diagnostic criteria, and characteristic brainstem and basal ganglia lesions on MRI—progressively confirmed NBD as the unifying diagnosis.

Given that recurrences persisted despite treatment and treatment with anti‐TNF (infliximab or adalimumab) was ruled out due to the differential diagnosis with demyelinating disease, management with anti‐CD20 (rituximab or ocrelizumab) was considered. He was discharged on rituximab, mycophenolate, tapering prednisone, and supplementation (calcium and vitamin D).

At the beginning of 2024, he had been receiving prednisone at a dose of 60 mg/day before he unilaterally decided to stop treatment; a few days later, he presented hetero‐aggression with inattention, memory lapses, and incoherent speech. He explained that “I was in a coma before the COVID‐19 pandemic; last night I discovered the names of my children. I am 25 years old, and it is 2003.” A CT scan showed hypodensity in the left thalamic region, ruling out an ischemic or hemorrhagic disorder. An MRI was performed, which showed a decrease in the size of the lesions in the pons‐midbrain, basal ganglia, and internal capsule.

Because of the confusional symptoms and restlessness, a consultation with psychiatry was requested, which assessed him for the first time. He was described as having expansive, hyperthymic contact with tachylalia and psychomotor restlessness. He was suspicious and distrustful. 10 mg of olanzapine and 1 mg of clonazepam were introduced, which was adjusted during the stay in the ward. When he was discharged, he was prescribed 7.5 mg of olanzapine and 0.5 mg of clonazepam. The rest of his usual treatment was reintroduced.

He stopped taking his medication again before the end of the first week after discharge and was admitted to the Short‐Stay Psychiatric Unit, where he stayed for 17 days. He presented with behavioral disturbance with significant hetero‐aggression towards objects and explained that “I have been bewitched; I have cameras in my house; they turn the lights on and off.” He appeared excited and hyperthymic, with unrealistic plans for the future. The diagnostic hypothesis was a manic episode secondary to BD (along with corticosteroid therapy).

During admission, he presented with dysarthric speech, global insomnia, attacks of aggression, disinhibition, and delusions of harm towards family members (poorly structured). He exhibited apraxia and parallel thinking (“I have to put blankets on the floor because if I touch the tiles, I'll burn myself”) and appeared euphoric and excited, with a lot of energy. Antipsychotic treatment was gradually introduced, and his usual medication was readjusted.

With the prescribed treatment, the psychopathology resolved, and the patient became very critical of the symptoms he had presented. He expressed some concern about how he will be received in his neighborhood (in a manic phase he went out into the street shouting and breaking car mirrors), but therapeutic leave was arranged and passed without incident. Upon observing improvement, the patient was discharged from the hospital with outpatient follow‐up. Treatment consisted of 12 mg paliperidone, 100 mg quetiapine, 2 mg Rivotril, 200 mg brivaracetam, 2000 mg mycophenolate mofetil, 60 mg prednisone, and 20 mg omeprazole.

The following month he was evaluated by neurology, which described clinical stability, although spastic hemiparesis with hyperreflexia persisted, with some gait lateralization. The same treatment was maintained.

A summary of the symptomatology and treatment chronology of the clinical case is provided in Table S1.

### 3.2 Systematic Review

Thirty‐three studies were included, comprising 34 cases of patients with NBD disease. Among the case reports included in our review, 11 were of low methodological quality (33.3%), 13 were medium quality (39.4%), and nine were high quality (27.3%) (Table 1).

**TABLE 1:** Analysis of the methodological quality of the clinical case reports.

Low quality	Medium quality	High quality
Chiba et al. (1986) [2] Haouala et al. (2019) [2] Hasbek et al. (2012) [2] Koçer et al. (2007) [2] Maner et al. (2011) [2] Mirone et al. (1998) [2] Orsucci (1996) [2] Taş et al. (2018) [2] Aoun et al. (2019) [1] Moreira et al. (2012) [1] Nkam and Cottereau (2006) [1]	Borson (1982) [5] Erdoğan et al. (2019) [5] Hariri et al. (2010) [5] Ogawa et al. (1976) [5] van Ham et al. (2014) [5] Aydin et al. (2002) [4] Deniz et al. (2009) [4] Kurikawa et al. (2004) [4] de Vries and van Vliet (2001) [3] Dolapoglu and Kahya (2023) [3] Karroumi et al. (2024) [3] Özdemir et al. (2004) [3] Tosto et al. (2013) [3]	de Berardis et al. (2013) [8] Alevizos et al. (2004) [7] Budman and Sarcevic (2002) [7] Shen et al. (2023) [7] Verim et al. (2006) [7] Calabrò et al. (2013) [6] Goolamali et al. (1976) [6] Patel et al. (2013) [6] Uhl et al. (1985) [6]

*Note*: The exact number of criteria that each study met is indicated in square brackets.

#### 3.2.1 General Characteristics of the Included Case Studies

Most publications came from Türkiye (*n* = 10; 30.3%), followed by Italy (*n* = 5; 15.1%), the United States (*n* = 4; 12.1%), and Japan (*n* = 3; 9.1%). There was considerable heterogeneity in age of onset, symptoms, therapeutic approach, and clinical course (Table S2). Beyond the country of origin of the publications, only a minority (*n* = 6; 17.6%) specified the country of origin of the cases. Half of them came from the Mediterranean basin (*n* = 3; 50%), with Morocco being the most represented country (*n* = 2; 33%). Males predominated (*n* = 22; 64.70%), and the mean age was 37.14 years (Table S2).

#### 3.2.2 Chronology of General and Neuropsychiatric Symptoms

The age of onset and the interval between the appearance of systemic and neuropsychiatric symptoms varied among the reviewed studies (Table S2).

General symptoms had appeared in only one case during childhood (Goolamali et al. 1976), and some had occurred in adolescence (Budman and Sarcevic 2002; Verim et al. 2006; Deniz et al. 2009; Patel et al. 2013). However, a large number of cases had onset between the ages of 20 and 30 years (Borson 1982; Uhl et al. 1985; de Vries and van Vliet 2001; Özdemir et al. 2004; Maner et al. 2011; de Berardis et al. 2013; Erdoğan et al. 2019; Shen et al. 2023; Karroumi et al. 2024) and, predominantly, between the ages of 30 and 50 years (Chiba et al. 1986; Aydin et al. 2002; Alevizos et al. 2004; Kurikawa et al. 2004; Koçer et al. 2007; Hariri et al. 2010; Moreira et al. 2012; Calabrò et al. 2013; Tosto et al. 2013 Aoun et al. 2019; Haouala et al. 2019). In no patients had the general symptoms begun after the age of 50 years, although in other cases, the age of onset was not specified (Ogawa et al. 1976; Orsucci 1996; Mirone et al. 1998; de Vries and van Vliet 2001; Nkam and Cottereau 2006; Hasbek et al. 2012; van Ham et al. 2014 Taş et al. 2018; Dolapoglu and Kahya 2023).

Neuropsychiatric symptoms had mostly appeared alongside the systemic onset (Aydin et al. 2002; Özdemir et al. 2004; Verim et al. 2006; Koçer et al. 2007; Deniz et al. 2009; Calabrò et al. 2013; Patel et al. 2013; Aoun et al. 2019; Haouala et al. 2019). Likewise, it was also common for symptoms to appear before 5 years (Chiba et al. 1986; Mirone et al. 1998; Hasbek et al. 2012; Moreira et al. 2012; Tosto et al. 2013) or even between 5 and 10 years of age, after the appearance of systemic symptoms (Uhl et al. 1985; Budman and Sarcevic 2002; Alevizos et al. 2004; Hariri et al. 2010; de Berardis et al. 2013; Shen et al. 2023; Karroumi et al. 2024). In a minority of cases, symptoms had appeared 10–20 years later (Borson 1982; Maner et al. 2011; Erdoğan et al. 2019), or even after 20 years (Goolamali et al. 1976; Kurikawa et al. 2004), although some studies did not provide these details (Ogawa et al. 1976; Orsucci 1996; de Vries and van Vliet 2001; Nkam and Cottereau 2006; van Ham et al. 2014; Taş et al. 2018; Dolapoglu and Kahya 2023).

#### 3.2.3 Neurological Manifestations

Regarding the description of the symptoms, neurological impairment consisting of cognitive impairment (Ogawa et al. 1976; Borson 1982; Mirone et al. 1998; de Vries and van Vliet 2001; Budman and Sarcevic 2002; Alevizos et al. 2004; Kurikawa et al. 2004; Koçer et al. 2007; Patel et al. 2013; Tosto et al. 2013; Aoun et al. 2019; Haouala et al. 2019), headache (Ogawa et al. 1976; Mirone et al. 1998; Kurikawa et al. 2004; Özdemir et al. 2004; Nkam and Cottereau 2006; Koçer et al. 2007; Patel et al. 2013; Haouala et al. 2019), paresis (Chiba et al. 1986; Aydin et al. 2002; Deniz et al. 2009; Maner et al. 2011; Hasbek et al. 2012; Calabrò et al. 2013; Haouala et al. 2019), hyperreflexia (Ogawa et al. 1976; Borson 1982; Aydin et al. 2002; Kurikawa et al. 2004; Verim et al. 2006; Hasbek et al. 2012; van Ham et al. 2014), speech disorders (Ogawa et al. 1976; Borson 1982; Aydin et al. 2002; Verim et al. 2006; Koçer et al. 2007; Deniz et al. 2009; de Berardis et al. 2013), gait disturbances (Alevizos et al. 2004; Verim et al. 2006; Koçer et al. 2007; Hasbek et al. 2012; van Ham et al. 2014), parkinsonism or other movement disorders (Ogawa et al. 1976; Budman and Sarcevic 2002; Kurikawa et al. 2004; Verim et al. 2006; Aoun et al. 2019), positive Babinski sign (de Vries and van Vliet 2001; Aydin et al. 2002; Verim et al. 2006), coordination disorders (Borson 1982; Hasbek et al. 2012), paresthesias (Calabrò et al. 2013; Shen et al. 2023), bladder and sphincter disorders (Hasbek et al. 2012; Aoun et al. 2019), seizures (Budman and Sarcevic 2002; Patel et al. 2013), confusional episodes and disorientation (Borson 1982; Aoun et al. 2019), oculomotor disorders (Ogawa et al. 1976; Aoun et al. 2019), visual disturbances (Aydin et al. 2002), encephalitis (Erdoğan et al. 2019) and dysphagia (Hasbek et al. 2012), among others, were detected (Table S2).

#### 3.2.4 Psychiatric Manifestations

At the psychiatric level, cases frequently presented psychopathology compatible with psychotic episodes, including delusions of persecution, somatic delusions, delusions of filiation, and megalomania, as well as self‐references, auditory, visual, and tactile hallucinations, thought disorganization, behavioral disorganization, neologisms, psychomotor agitation, thought withdrawal, social isolation, impoverishment of speech, perplexity, and affective flattening (Ogawa et al. 1976; Borson 1982; Chiba et al. 1986; Mirone et al. 1998; de Vries and van Vliet 2001; Kurikawa et al. 2004; Özdemir et al. 2004; Nkam and Cottereau 2006; Verim et al. 2006; Deniz et al. 2009; Moreira et al. 2012; Calabrò et al. 2013; Patel et al. 2013; Taş et al. 2018; Erdoğan et al. 2019; Haouala et al. 2019; Dolapoglu and Kahya 2023; Karroumi et al. 2024). In some cases, schizophrenia (de Berardis et al. 2013; Goolamali et al. 1976) or organic delusional disorder (Uhl et al. 1985) had been diagnosed (Table S2).

Depressive affective symptoms including sadness, anhedonia, abulia, social isolation, and suicidal thoughts and attempts were also described (Borson 1982; Uhl et al. 1985; Orsucci 1996; Aydin et al. 2002; Budman and Sarcevic 2002; Alevizos et al. 2004; Kurikawa et al. 2004; Özdemir et al. 2004; Nkam and Cottereau 2006; Koçer et al. 2007; Hariri et al. 2010; Tosto et al. 2013; Aoun et al. 2019; Haouala et al. 2019; Shen et al. 2023). Conversely, manic or hypomanic episodes were also reflected with euphoria, psychomotor agitation, verbosity, insomnia, excessive spending, and disinhibition (Orsucci 1996; Aydin et al. 2002; Alevizos et al. 2004; Nkam and Cottereau 2006; Koçer et al. 2007; Hariri et al. 2010; Hasbek et al. 2012; van Ham et al. 2014; Haouala et al. 2019; Shen et al. 2023), resulting in a diagnosis of bipolar disorder in some cases (Maner et al. 2011; Moreira et al. 2012) (Table S2).

As for other psychopathological conditions, changes in personality or behavior were described, with strange behavior, mutism, antisocial behavior, and aggression (Ogawa et al. 1976; de Vries and van Vliet 2001; Calabrò et al. 2013); obsessive‐compulsive contamination symptoms (Budman and Sarcevic 2002; Patel et al. 2013); and somatoform disorders (Calabrò et al. 2013) (Table S2).

#### 3.2.5 Neuroimaging and Neurophysiological Findings

Imaging tests reflected cerebellar (Alevizos et al. 2004; Koçer et al. 2007), cortical (Borson 1982; Verim et al. 2006), hippocampal (de Vries and van Vliet 2001), temporal (de Vries and van Vliet 2001), and brainstem (Koçer et al. 2007) atrophy. Ventricular dilation (Borson 1982; de Vries and van Vliet 2001; Alevizos et al. 2004) and subarachnoid dilation (Alevizos et al. 2004) were also described (Table S2).

Structural lesions were found in the internal capsule (de Vries and van Vliet 2001; Deniz et al. 2009; Calabrò et al. 2013), basal ganglia (Kurikawa et al. 2004; Aoun et al. 2019), amygdala (Deniz et al. 2009; Calabrò et al. 2013), lateral and longitudinal sinuses (Nkam and Cottereau 2006; Karroumi et al. 2024), anterior cingulate gyrus (Calabrò et al. 2013), hippocampal gyrus (Deniz et al. 2009), capsulostriate regions (de Berardis et al. 2013), and meningeal structures (Özdemir et al. 2004). Lesions were also observed in the brainstem (Deniz et al. 2009; Hasbek et al. 2012) and more specifically in the midbrain (de Berardis et al. 2013; van Ham et al. 2014) and the pons (Aydin et al. 2002; Tosto et al. 2013) Table S2). Lesions in the white matter were also described (de Vries and van Vliet 2001; Kurikawa et al. 2004; Özdemir et al. 2004; Hasbek et al. 2012; Moreira et al. 2012; Patel et al. 2013), and on several occasions, demyelination (van Ham et al. 2014; Aoun et al. 2019) and gliotic lesions (van Ham et al. 2014; Verim et al. 2006) were found. In addition, thrombotic lesions were found in the dural sinuses (Patel et al. 2013) (Table S2).

In functional neuroimaging tests, alterations were detected in the EEG in the form of temporal hypoactivity (de Vries and van Vliet 2001; Verim et al. 2006), frontal hypoactivity (Özdemir et al. 2004), and cortical hypoactivity (van Ham et al. 2014). Alterations were described in alpha waves (Ogawa et al. 1976; Chiba et al. 1986), beta waves (Goolamali et al. 1976), and theta and delta waves (de Vries and van Vliet 2001). Abnormal findings were also reflected in single‐photon emission computed tomography (SPECT) in the form of hypoperfusion in the frontal lobes (Mirone et al. 1998; Budman and Sarcevic 2002; van Ham et al. 2014) and temporal lobes (Budman and Sarcevic 2002; van Ham et al. 2014), as well as in the cortical area and caudate nucleus (Budman and Sarcevic 2002) (Table S2). However, numerous studies showed normal results in the various complementary tests performed.

#### 3.2.6 Treatments Used

Most of the reviewed studies had used systemic corticosteroids for somatic treatment (Goolamali et al. 1976; Ogawa et al. 1976; Uhl et al. 1985; Chiba et al. 1986; Orsucci 1996; Mirone et al. 1998; de Vries and van Vliet 2001; Aydin et al. 2002; Budman and Sarcevic 2002; Alevizos et al. 2004; Kurikawa et al. 2004; Nkam and Cottereau 2006; Koçer et al. 2007; Deniz et al. 2009; Maner et al. 2011; Hasbek et al. 2012; Moreira et al. 2012; de Berardis et al. 2013; Patel et al. 2013; Tosto et al. 2013; van Ham et al. 2014; Haouala et al. 2019; Dolapoglu and Kahya 2023; Shen et al. 2023), but some had used other anti‐inflammatory treatments such as colchicine (Orsucci 1996; Özdemir et al. 2004; Alevizos et al. 2004; Nkam and Cottereau 2006; Koçer et al. 2007; Hariri et al. 2010; Hasbek et al. 2012; van Ham et al. 2014; Dolapoglu and Kahya 2023) or NSAIDs (Alevizos et al. 2004; Dolapoglu and Kahya 2023) (Table S2).

Immunomodulatory treatments such as azathioprine (Budman and Sarcevic 2002; Nkam and Cottereau 2006; Maner et al. 2011; Hasbek et al. 2012; de Berardis et al. 2013; van Ham et al. 2014; Erdoğan et al. 2019; Haouala et al. 2019; Dolapoglu and Kahya 2023; Karroumi et al. 2024), cyclosporine (Mirone et al. 1998; de Vries and van Vliet 2001; Hasbek et al. 2012; Erdoğan et al. 2019; Shen et al. 2023), cyclophosphamide (Nkam and Cottereau 2006; de Berardis et al. 2013; Tosto et al. 2013; Aoun et al. 2019), mycophenolate (Budman and Sarcevic 2002; Erdoğan et al. 2019), mercaptopurine (Ogawa et al. 1976), or chlorambucil (Uhl et al. 1985) had also been widely used (Table S2).

In some cases, biological therapies such as infliximab (Patel et al. 2013) and other complementary drugs such as anticoagulants or antiplatelet agents (Dolapoglu and Kahya 2023; Nkam and Cottereau 2006; Özdemir et al. 2004; Patel et al. 2013), immunoglobulins (Özdemir et al. 2004), methotrexate (de Berardis et al. 2013), hydroxychloroquine (Budman and Sarcevic 2002), levamisole (Orsucci 1996), or antiepileptics such as lacosamide (Erdoğan et al. 2019) or lamotrigine (Patel et al. 2013) had also been used (Table S2).

To treat psychiatric symptoms, antipsychotics such as risperidone (de Vries and van Vliet 2001; Budman and Sarcevic 2002; Özdemir et al. 2004; Nkam and Cottereau 2006; Deniz et al. 2009; de Berardis et al. 2013; Taş et al. 2018; Haouala et al. 2019; Karroumi et al. 2024), haloperidol (Borson 1982; Uhl et al. 1985; Budman and Sarcevic 2002; Nkam and Cottereau 2006; Hariri et al. 2010; de Berardis et al. 2013; Patel et al. 2013; Erdoğan et al. 2019), olanzapine (Alevizos et al. 2004; Hariri et al. 2010; Moreira et al. 2012; de Berardis et al. 2013; van Ham et al. 2014; Erdoğan et al. 2019), quetiapine (Verim et al. 2006; Hasbek et al. 2012; Taş et al. 2018; Shen et al. 2023), aripiprazole (de Berardis et al. 2013; Dolapoglu and Kahya 2023), zuclopenthixol (Alevizos et al. 2004; Hasbek et al. 2012), clozapine (de Berardis et al. 2013), lurasidone (Shen et al. 2023), ziprasidone (de Berardis et al. 2013), amisulpride (Calabrò et al. 2013), clothiapine (van Ham et al. 2014), pipamperone (van Ham et al. 2014), and phenothiazines (Goolamali et al. 1976), more specifically perphenazine (Alevizos et al. 2004), had been used (Table S2).

Selective Serotonin Reuptake Inhibitors (SSRI) antidepressant drugs such as sertraline (Verim et al. 2006; Koçer et al. 2007; Moreira et al. 2012; Tosto et al. 2013; Taş et al. 2018; Dolapoglu and Kahya 2023), escitalopram (Haouala et al. 2019), and paroxetine (Budman and Sarcevic 2002); tricyclics (Borson 1982), more specifically amitriptyline (Alevizos et al. 2004; Haouala et al. 2019) and desipramine (Uhl et al. 1985); tetracyclics such as maprotiline (Kurikawa et al. 2004) and mianserin (Özdemir et al. 2004); and dual drugs such as venlafaxine (Calabrò et al. 2013; Shen et al. 2023) had also been used. Antidepressants had been used in some cases, although they were not detailed (Aoun et al. 2019; Goolamali et al. 1976) (Table S2).

Some of the studies had used mood stabilizers such as sodium valproate (Budman and Sarcevic 2002; Alevizos et al. 2004; Hariri et al. 2010; Hasbek et al. 2012; Moreira et al. 2012; van Ham et al. 2014; Haouala et al. 2019), lithium (Aydin et al. 2002; Alevizos et al. 2004; Hariri et al. 2010; Shen et al. 2023), or carbamazepine (Budman and Sarcevic 2002) (Table S2). In terms of anxiolytics, clonazepam (Budman and Sarcevic 2002; Nkam and Cottereau 2006; Hariri et al. 2010), diazepam (Borson 1982; Kurikawa et al. 2004), lorazepam (Karroumi et al. 2024), alprazolam (Nkam and Cottereau 2006), lormetazepam (van Ham et al. 2014), or etizolam (Kurikawa et al. 2004) had been used, while hypnotic drugs such as zopiclone (Kurikawa et al. 2004) or zolpidem (Nkam and Cottereau 2006) had been used on other occasions (Table S2).

In certain patients, other interventions such as electroconvulsive therapy (Goolamali et al. 1976) or psychotherapy (Borson 1982; Orsucci 1996) had also been used. Finally, some studies did not specify the psychiatric treatment administered (Ogawa et al. 1976; Chiba et al. 1986; Mirone et al. 1998; Maner et al. 2011) (Table S2).

#### 3.2.7 Progression and Prognosis

The prognosis in the different studies was variable. The most frequent outcome was a partial improvement of the symptoms, using corticosteroids both as monotherapy (Goolamali et al. 1976; Chiba et al. 1986; Aydin et al. 2002; Deniz et al. 2009) and in combination with colchicine (Koçer et al. 2007), infliximab (Patel et al. 2013), cyclosporine (Shen et al. 2023), or 6‐mercaptopurine (Ogawa et al. 1976) (Table S2).

In a minority of cases, complete remission of neuropsychiatric symptoms had occurred when using monotherapy with risperidone (Deniz et al. 2009), lamotrigine (Patel et al. 2013), lithium (Shen et al. 2023), sertraline (Tosto et al. 2013), quetiapine (Verim et al. 2006), or desipramine (Uhl et al. 1985). However, in most cases, polytherapy strategies had been used. The most frequent treatment combinations were antipsychotics with mood stabilizers (Alevizos et al. 2004; Hasbek et al. 2012; van Ham et al. 2014) and antipsychotics with antidepressants (Borson 1982; Budman and Sarcevic 2002; Özdemir et al. 2004; Calabrò et al. 2013; Taş et al. 2018; Dolapoglu and Kahya 2023). After this, the most common treatment had been a combination of several antipsychotics (de Berardis et al. 2013; Erdoğan et al. 2019), frequently associated with benzodiazepines (Nkam and Cottereau 2006; Karroumi et al. 2024) (Table S2).

Other less frequent combinations included mood stabilizers and antidepressants (Moreira et al. 2012) and even electroconvulsive therapy combined with antipsychotics and antidepressants in complex cases (Goolamali et al. 1976). In one specific case, the symptoms were improved through lifestyle changes (Orsucci 1996) (Table S2).

Conversely, there were cases in which no improvement was achieved at any time (de Vries and van Vliet 2001) or in which an overall progressive clinical deterioration was observed (Budman and Sarcevic 2002; Kurikawa et al. 2004), sometimes associated with persistent negative clinical symptoms (Nkam and Cottereau 2006; Karroumi et al. 2024) or learning difficulties and temporal disorientation (Koçer et al. 2007). In addition, in some patients, cognitive impairment appeared, which was sometimes persistent (Ogawa et al. 1976; Borson 1982; de Vries and van Vliet 2001) or could be reversed with drugs such as memantine (Koçer et al. 2007) (Table S2).

In other scenarios, symptomatic relapses appeared due to poor compliance (Haouala et al. 2019), or manic switches had been induced after treatment with antidepressants such as amitriptyline (Haouala et al. 2019) or hypomanic switches with venlafaxine (Shen et al. 2023). One case report also described a patient that had died (Aoun et al. 2019) (Table S2).

### 3.3 Sensitivity Analysis

By removing the 11 (33.3%) low‐quality studies and synthesizing the findings of the medium and high‐quality studies, the key descriptive patterns found remained: 65% men, parenchymal predominance, and psychotic/manic symptoms.

## 4 Discussion

The clinical case presented is of great interest, as it illustrates the NBD paradigm. Furthermore, given the patient's characteristics, it serves to justify this systematic review of the different psychiatric presentations of NPBD. Regarding the clinical case presented here, it concerns a 39‐year‐old Moroccan man who began to show general symptoms of BD. In our systematic review, most of the articles came from countries in the Mediterranean basin, such as Türkiye, Italy, or Morocco, the latter being the most frequent origin among the cases in which nationality was specified. The review also showed that the average age of onset of BD symptoms was 37.1 years, moving away from the classic consideration that the onset of BD occurs in the second to third decade of life and approaching the age of the patient described in the clinical case.

One year later, the patient presented with neurological symptoms suggestive of NBD, with headache being the sentinel symptom. Specifically, the symptoms of his NBD reflected CNS involvement with parenchymal lesions—hemiparesis, hypoesthesia, cognitive impairment, disorientation, dysmetria, or spasticity, among others (Talarico et al. 2015; Ali and Das 2018; Beça and Espinosa 2021). Furthermore, 3 years later, the patient developed NPBD, consisting of symptoms already described in other cases such as euphoria, pressive speech, flight of ideas, tendency toward distraction, and motor hyperactivity (Bozikas et al. 2004), with hyperphagia and behavioral alterations (Nakano et al. 2004). Interestingly, this occurred following abrupt discontinuation of corticosteroid treatment, something already described in previous studies (Demirci et al. 2015; Hibberd et al. 2018).

With regard to the pathochronia, although the scientific literature reports cases in which neuropsychiatric symptoms precede any other manifestation (Nakano et al. 2004), our systematic review shows that it usually appears at the same time or within the first 5 years after the onset of somatic symptoms of BD, with headache being one of the most common symptoms. Regarding aetiopathogenesis, the relationship between parenchymal lesions and the onset of various neuropsychiatric symptoms is well known (Talarico et al. 2015; Ali and Das 2018; Beça and Espinosa 2021), which may explain the aforementioned temporal pattern of the disease.

In this regard, our systematic review found that a large proportion of NPBD cases showed structural abnormalities in neuroimaging tests, which may support the hypothesis that the onset of neuropsychiatric symptoms depends on the area of the brain affected. This could contribute to justifying the variety of psychopathological presentations we recorded: from psychotic episodes (Mirone et al. 1998; Moreira et al. 2012) to obsessive (Budman and Sarcevic 2002) to depressive symptoms (Uhl et al. 1985; Kurikawa et al. 2004) or manic symptoms, the latter being among the most frequent presentations (da Mota Freitas and Guerra 2023). Specifically, our investigation suggests that psychiatric manifestations in NPBD are mainly associated with disruption of fronto‐subcortical and limbic networks. Psychotic symptoms were most frequently linked to involvement of the basal ganglia, capsulostriatal regions, and limbic structures. Affective manifestations, including depression or manic presentations, were often reported in association with white matter lesions, cerebellar atrophy, and brainstem involvement. In contrast, behavioral disorders and obsessive–compulsive symptoms were commonly associated with fronto‐striatal alterations. The correlation between lesions and psychiatric phenotype is summarized in Table S3.

Nevertheless, it remains unclear whether these structural findings directly generate psychiatric syndromes, whether they reflect parallel disease processes, or whether additional biological and psychosocial factors modulate their clinical expression. Furthermore, there is currently a debate about whether psychiatric involvement in BD is a primary inflammatory manifestation or a secondary phenomenon resulting from it (Talarico et al. 2018). In this context, a conceptual inflammatory–neurotransmitter model may help to better explain the emergence of psychiatric manifestations in NBD.

According to this framework, systemic immune dysregulation associated with BD can trigger neuroinflammatory processes within the CNS, particularly in parenchymal forms of the disease. Elevated levels of pro‐inflammatory cytokines, including IL‐6, TNF‐α, and other Th17‐related mediators, may compromise blood–brain barrier integrity and alter monoaminergic transmission, glutamatergic signaling, neuroplasticity, ory, but is stric and hypothalamic–pituitary–adrenal axis regulation (Liu et al. 2026), involved in affective and psychotic disorders (Miller and Raison 2016; Islam et al. 2023). Within this perspective, observed structural lesions may interact with cytokine‐mediated neurochemical dysregulation (Felger and Miller 2012), ultimately contributing to the development of psychiatric symptoms, particularly in patients with sustained systemic and neuroinflammatory activity.

It should be noted that some authors have pointed out that there is no relationship between neuropsychiatric clinical findings and parenchymal lesions on MRI (Monastero et al. 2004), and in our review, several studies showed normal results in the various neuroimaging tests performed. Even more, other studies found that the presence of psychiatric disorders does not appear to be related to neurological involvement in a patient's medical history but is strictly related to disease activity, suggesting that NPBD may be an intrinsic aspect of BD (Talarico et al. 2018). Conversely, it has been observed that mental and behavioral deterioration continues to progress even during periods free of recurrence. This suggests that the disease is an active, continuous process at the CNS level (Bozikas et al. 2015).

**FIGURE 2:**
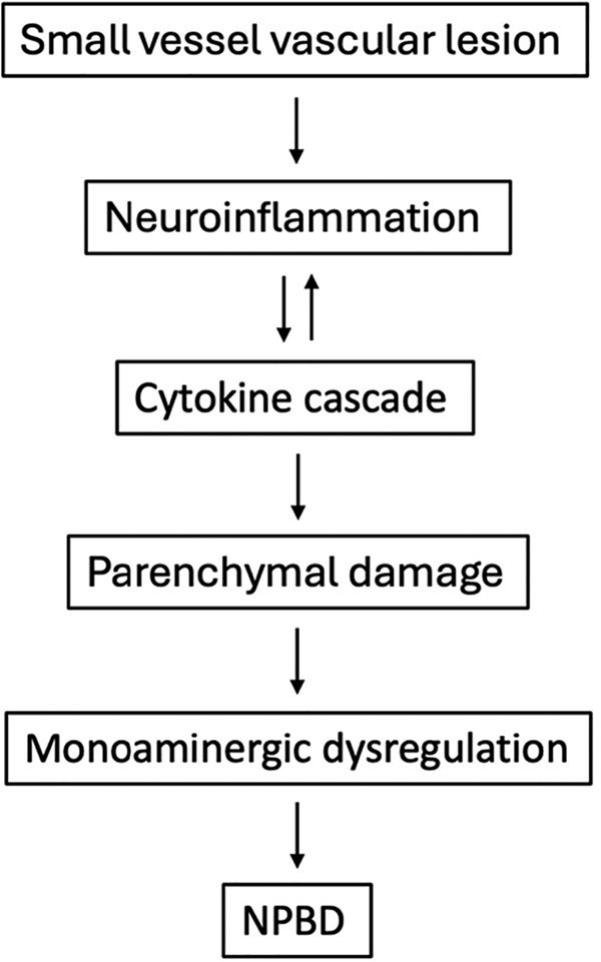
Schematic of the biphasic model.

This could suggest the hypothesis of a biphasic model (Figure 2) in which small vessel vascular lesions in BD induce neuroinflammation and subsequent monoaminergic dysregulation through a cytokine cascade, culminating in clinical manifestations. Thus, it would initially give rise to alterations that go unnoticed at the macroscopic or symptomatic level but may ultimately lead to parenchymal damage as part of a continuous process in which psychiatric symptoms emerge in different forms depending on the stage of the disease (Alevizos et al. 2004; van Ham et al. 2014). This model should be considered a theoretical framework that requires confirmation in longitudinal and controlled studies, since other variables such as psychosocial burden must be assessed.

**FIGURE 3:**
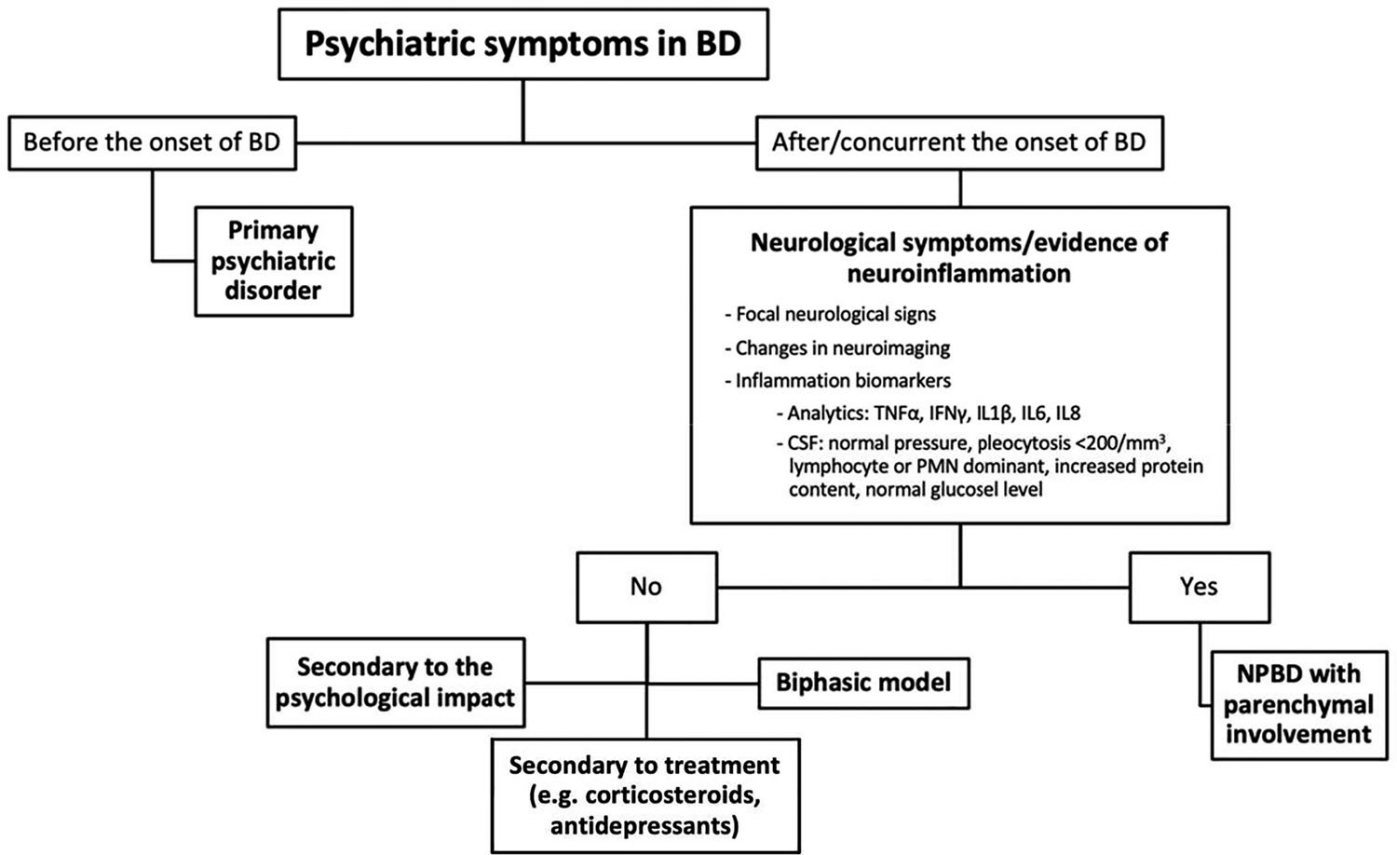
Algorithm for the etiopathogenesis of psychiatric symptoms in BD.

In line with this biphasic theory, in our clinical case, manic symptoms arose in the absence of an obvious neurological focus and following the abrupt discontinuation of corticosteroid treatment, which may have contributed to exacerbated parenchymal neuroinflammation and monoaminergic dysregulation in certain areas of the brain related to these symptoms, as described in previous studies (Demirci et al. 2015; Hibberd et al. 2018). This possibility should be considered, as corticosteroids are known to induce affective symptoms, even during withdrawal (Gostoli et al. 2025). In this regard, the biphasic theory does not exclude alternative or complementary explanations related to the appearance of psychiatric symptoms, thereby making it difficult to establish a single, definitive aetiopathogenesis. For this reason, the following algorithm is proposed (Figure 3), which may facilitate the identification of the etiopathogenesis and the classification of psychopathological presentations in accordance with recognized diagnostic frameworks, such as the DSM‐5 or the ICD‐11.

Thus, the suspicion of NPBD should be based on an objective diagnosis of BD; a physical examination to identify neurological symptoms or the support of objective evidence of neurological involvement; a psychopathological assessment to identify the psychiatric symptoms and their timing relative to the onset of BD; as well as a thorough differential diagnosis (Table 2).

**TABLE 2:** Diagnostic framework for NPBD.

Criterion	Description
**1. Diagnosis of Behçet's disease**	Based on objective criteria such as the ICBD.
**2. Presence of neuropsychiatric syndrome**	**A. Neurological involvement (≥ 1 required)** – **Symptoms suggestive of CNS involvement** 1) Parenchymal symptoms 2) Non‐parenchymal symptoms 3) Both parenchymal and non‐parenchymal symptoms. – **Objective evidence of CNS involvement** 1) MRI or CT abnormalities, CSF findings, or neurophysiological abnormalities. **B. Psychiatric manifestations** – At least one related to psychosis, mood disorders, obsessive‐compulsive symptoms, behavioral or personality changes, etc.
**3. Temporal association**	Neuropsychiatric symptoms must occur after or concurrent with Behçet's disease diagnosis.
**4. Exclusion criteria**	– Primary psychiatric disorders – Drug‐induced states (e.g., corticosteroids) – Infections (e.g., Herpes simplex encephalitis) – Other neuroinflammatory diseases (e.g., Multiple sclerosis)

Abbreviations: CNS, central nervous system; CSF, cerebrospinal fluid; CT, computed tomography; ICBD, International Criteria for Behçet's Disease; MRI, magnetic resonance imaging.

As in the patient case described, treatment should include an autoimmune approach with corticosteroids, immunosuppressants, interferon‐alpha, and TNF inhibitors (Lavalle et al. 2024) for the treatment of BD. A simultaneous psychiatric approach is essential, including the use of antipsychotics (olanzapine, quetiapine) (van Ham et al. 2014; Bozikas et al. 2015), mood stabilizers (carbamazepine, valproate) (Talarico et al. 2015), etc., depending on the psychiatric condition present. Indeed, previous studies support the combination of psychiatric and anti‐inflammatory treatment in bipolar disorder secondary to BD (da Mota Freitas and Guerra 2023). Even more, this therapeutic approach was reported in most of the studies included in our analysis. Based on this evidence, we propose a simplified therapeutic decision algorithm for managing NPBD (Figure S1).

Our findings should be interpreted with caution given the methodological limitations inherent to case‐based literature, including incomplete clinical characterization and limited follow‐up. The risk of bias assessment identified recurrent weaknesses across several domains—particularly causality assessment, completeness of case reporting, and duration of follow‐up—which may affect the reliability of the reported neuroimaging–psychiatric associations. In contrast, fewer concerns were identified in other domains, such as selection and ascertainment. Another potential limitation is that one‐third of the studies are of low quality. However, sensitivity analysis shows that the results are robust, as they do not change when these low‐quality studies are removed from the synthesis.

Our systematic review reinforces not only the relationship between BD and bipolar disorder but also with other psychiatric disorders or symptoms. However, this relationship should be understood primarily as an association observed in case‐based literature rather than as proof of direct causality. Although various etiopathogenic mechanisms have been hypothesised, no conclusive research is available. Thus, it is essential to investigate the biological basis underlying the onset of neuropsychiatric symptoms in BD, identify possible common pathophysiological mechanisms, and weigh the importance of external factors such as pharmacological treatments and psychosocial stress associated with chronic systemic disease.

## 5 Conclusion

The case presented exemplifies the clinical and etiopathogenic complexity of BD with neuropsychiatric involvement. Its evolution—from the appearance of systemic symptoms to the development of neurological and, subsequently, psychiatric manifestations—reflects the multiphasic course described in the literature, characterized by progressive involvement of the CNS. The review of published cases confirms that NPBD can present through a wide spectrum of psychopathological alterations, ranging from affective and psychotic disorders to personality and behavioral disorders.

The correlation between parenchymal lesions and the appearance of psychiatric symptoms, observed in most of the reviewed studies and in the described case, reinforces the hypothesis of a common neurobiological substrate mediated by inflammatory and immunological mechanisms. In this sense, the possible existence of a biphasic model—where the initial vascular involvement evolves into a neurochemical and structural dysfunction—offers a coherent model.

Furthermore, the appearance of psychiatric symptoms after the abrupt discontinuation of corticosteroid treatment highlights the vulnerability of the neuroinflammatory balance and the importance of carefully adjusted pharmacological management. Taken together, these findings underscore the need for an interdisciplinary approach that integrates neurological, psychiatric, and immunological perspectives and for future research aimed at clarifying the pathophysiological mechanisms involved in NPBD in order to optimize early diagnosis and personalized therapeutic strategies.

## Author Contributions

**Jorge Renau**: conceptualization, investigation, methodology, writing – original draft, writing – review and editing. **Iván Echeverria**: conceptualization, investigation, methodology, funding acquisition, supervision, writing – original draft, writing – review and editing. **Ana Benito**: methodology, formal analysis, supervision, writing – review and editing. **Elena Sesé**: investigation, writing – review and editing. **María Vicenta Lucas‐Miralles**: writing – review and editing. **Marc Peraire**: conceptualization, investigation, methodology, supervision, writing – original draft, writing – review and editing.

## Ethics Statement

This study includes a systematic review and a clinical case report. Ethical approval was not required for the systematic review. For the clinical case, written informed consent was obtained from the patient for publication.

## Funding

This study received funding from Universidad Cardenal Herrera–CEU, CEU Universities (INDI 25/26). The funders were not involved in the study design, data collection, analysis, interpretation of data, the writing of this article, or the decision to submit it for publication.

## Conflicts of Interest

The authors declare no conflicts of interest.

## Supporting information




**Supplementary Material**: brb371593‐sup‐0001‐SuppMat.docx

## Data Availability

The data that support the findings of this study are available from the corresponding author upon reasonable request.
