# Supplementary material for: Neuro‐Behçet's Disease and Psychiatric Disorders: From a Case Report to a Systematic Review
Source: Brain Behav. 2026 Jul 23;16(7):e71593. doi: 10.1002/brb3.71593 (PMC13396884; doi:10.1002/brb3.71593)
Supplement: Supplementary file 1 — Supplementary Material: brb371593‐sup‐0001‐SuppMat.docx [file BRB3-16-e71593-s001.docx]

**Table 1S.** Symptomatology and treatment chronology.

| **Date** | **Clinical event** | **Diagnostic findings** | **Treatment** |
| --- | --- | --- | --- |
| Jun 2021 | Recurrent oral ulcers |  | Symptomatic treatment |
| Oct 2022 | Seizure, aphasia, tetraparesis | MRI: temporal edema + bulbopontine lesion  CSF: pleocytosis | Ceftriaxone, acyclovir, high-dose corticosteroids |
| May 2023 | Headache, diplopia, hemiparesis | MRI: pontine lesion with ring enhancement | Prednisone 50 mg, mycophenolate mofetil 500 mg, trimethoprim-sulfamethoxazole 400/80 mg |
| Nov 2023 | Neurological relapse (right facio-brachio-crural hemiparesis and visual loss) | MRI: new pontine and basal ganglia lesions | Rituximab, prednisone, mycophenolate, supplementation (calcium and vitamin D) |
| Early 2024 | Treatment discontinuation | CT: hypodensity in the left thalamic region  MRI: decreased lesions in the pons-midbrain, basal ganglia, and internal capsule | Olanzapine 10 mg, clonazepam 1 mg |
| 2024 | Manic episode (Admision to Short-Stay Psychiatric Unit) | Clinical psychiatric assessment | Paliperidone 12 mg, quetiapine 100 mg, clonazepam 2 mg, brivaracetam 200 mg, mycophenolate mofetil 2000 mg, prednisone 60 mg, omeprazole 20 mg |

**Table S2.** Results of the studies analysed.

| **Author (year)** | **Country** | **Patient characteristics** | **History of Behçet’s disease** | **Neuropsychiatric symptoms** | **Imaging tests** | **Management and treatment** |
| --- | --- | --- | --- | --- | --- | --- |
| Alevizos et al. (2004) | Greece | - ♀, 62 years old (nationality not indicated).  - No relevant clinical history. | **- Symptoms:** Onset at age 38 with recurrent iridocyclitis, orogenital ulcers, and erythema nodosum.  **- HLA-B5:** (+) | **- Neurological:** At 60 years old, gait instability and cognitive impairment.  **- Psychiatric:** Diagnosis of bipolar disorder type 1 at age 44 after a manic episode (euphoria, psychomotor agitation, verbosity, insomnia, and excessive spending). Subsequently, manic episodes preceded by depressive episodes and rapid cycling. | **- Brain CT and MRI**: Cerebellar atrophy, mild ventricular enlargement, and subarachnoid space enlargement. | **- General:** Colchicine 100 mg/day, NSAIDs (diclofenac 75–150 mg/day), corticosteroids, with remission of the clinical symptoms of BD.  **- Psychiatric:** Over the years, zuclopenthixol, perphenazine, amitriptyline, and lithium had been used. Finally, mood stabilisation was achieved with carbamazepine 600 mg/day, sodium valproate 1,500 mg/day, and olanzapine 5 mg/day. |
| Aoun et al. (2019) | Tunisia | - ♂, 50 years old (nationality not indicated).  - No relevant clinical history. | **- Symptoms:** Onset and diagnosis of BD at age 50 with uveitis along with psychiatric symptoms. | **- Neurological:** At 50 years of age, cognitive impairment, disorientation, parkinsonism, oculomotor and bladder/sphincter disorders.  **- Psychiatric:** Onset at age 50 with a depressive episode (sadness, anhedonia, and abulia). | **- Brain MRI:** Demyelinating lesions in the basal ganglia. | **- General:** Cyclophosphamide. The patient died 6 months after diagnosis.  **- Psychiatric:** Antidepressants (not specified). |
| Aydin et al. (2002) | Türkiye | - ♂, 35 years old (nationality not indicated).  - No relevant clinical history. | **- Symptoms:** Onset at age 35 with lingual ulcer and psychiatric symptoms. Later, orogenital ulcers.  **- Pathergy test:** (+).  **- CSF:** Increased protein and pleocytosis. | **- Neurological:** At age 35 years, tetraparesis, gait disorder, dysarthria, diplopia, paresis of the abducens and facial nerves, hyperreflexia, Babinski +, and nuchal rigidity.  **- Psychiatric:** Hypomanic episode at age 35 (insomnia, behavioural disinhibition, euphoria, and excessive spending). Subsequently, a depressive episode with irritability. | **- Cranial CT scan:** No alterations.  **- Brain MRI:** Hypointense oedematous lesion in the pons. | **- General:** Methylprednisolone 1 mg/kg/day with neurological and psychiatric clinical improvement.  **- Psychiatric:** Lithium 600 mg/day without clinical response. |
| Borson (1982) | United States | - ♂, 42 years old (nationality not indicated).  - No relevant clinical history. | **- Symptoms:** Onset at age 25 with recurrent orogenital ulcers, joint effusions, and vasculitis. Diagnosis of BD 10 years later.  **- CSF:** Pleocytosis and increased protein. | **- Neurological:** At 39 years old, hyperreflexia, unilateral Babinski. Subsequently, incoordination, cognitive impairment, confusional episodes, dysarthria, and dysmetria.  **- Psychiatric:** Diagnosis of adjustment disorder at age 35 (depressed mood, insomnia, and suicidal thoughts). Subsequently, several depressive episodes and one psychotic episode with paranoid delusions. | **- CT scan:** Cortical atrophy, symmetrical ventricular enlargement. | **- Psychiatric:** Initially, psychotherapy and diazepam. Subsequently, tricyclic antidepressants were used with a good response in terms of the depressive episodes, and haloperidol 2 mg/day with improvement of the psychotic symptoms. Cognitive decline persisted. |
| Budman & Sarcevic (2002) | United States | - ♂, 22 years old (nationality not indicated).  - History of recurrent bacterial and viral infections in childhood and adolescence. | **- Symptoms:** Onset at age 12 with epistaxis and oral ulcers. Subsequently, genital ulcers, painful lymphadenopathy, uveitis, arthralgia, fever, sore throat, and vesicular eruptions developed. Diagnosis of BD 7 years later.  **- CSF:** Negative culture. | **- Neurological:** Seizures, dyskinesias, myoclonus, and chorea. Progressive cognitive decline.  **- Psychiatric:** From age 20, emotional lability and disinhibition, severe contamination OCD (after starting an immunosuppressant), with accentuated physical and oral tics. | **- SPECT:** Mild diffuse cortical hypoactivity. Perfusion defects in the left frontotemporal junction, tail of the left basal ganglia, and floor of the right frontal lobe.  **- Brain MRI:** No alterations.  **- EEG:** No alterations. | **- General:** Prednisone, azathioprine, mycophenolate, dapsone, hydroxychloroquine, nizatidine, diphenhydramine, and metoprolol.  **- Psychiatric**: Carbamazepine, sodium valproate, clonazepam, and haloperidol. Improvement with risperidone and paroxetine, although overall clinical deterioration occurred. |
| Calabrò et al. (2013) | Italy | - ♀, 31 years old (nationality not indicated).  - No relevant clinical history. | **- Symptoms:** Onset at age 31, with suspicion of NBD by diagnosis of exclusion. Subsequently, recurrent genital ulcers.  **- Pathergy test:** (+) | **- Neurological:** Weakness in upper limbs, paresthesias, and mild hemiparesis.  **- Psychiatric:** Onset at age 31 with behavioural disturbances, excessive anxiety, loss of appetite, and visual and auditory hallucinations. Diagnosis of somatoform disorder. | **- Brain CT scan:** No alterations.  **- MRI (FLAIR):** Hyperintensities in the right tonsil, anterior cingulate gyrus, and posterior limb of the left internal capsule.  **- EEG:** No alterations. | **- General:** Colchicine 1 mg/day and aspirin 100 mg/day.  **- Psychiatric:** Venlafaxine 75 mg/day and amisulpride 50 mg/day, with improvement in a few weeks. |
| Chiba et al. (1986) | Japan | - ♀, 44 years old, originally from Japan.  - No relevant clinical history. | **- Symptoms:** Onset at age 42 with oral ulcers along with psychiatric symptoms. Later, genital ulcers, and pyoderma developed. Diagnosis of BD 2 years later.  **- CSF:** Mild lymphocytic pleocytosis.  **- Laboratory analytics:** Mild leukocytosis, elevated erythrocyte sedimentation rate (ESR). | **- Neurological:** At 45 years old, mild hemiparesis in the right limbs.  **- Psychiatric:** Onset at age 44 with emotional lability, tactile and visual hallucinations, delirium and stereotypies. Subsequently, emotional indifference and memory impairment. | **- Brain CT scan:** No alterations.  **- EEG:** Poor and irregular alpha activity, mild diffuse slowing. | **- General:** Prednisolone 60 mg/day in a tapering regimen, with improvement of psychiatric symptoms and normalisation of analytical and CSF findings. |
| De Berardis et al. (2013) | Italy | - ♂, 37 years old (nationality not indicated)  - No relevant clinical history. | **- Symptoms:** Onset and diagnosis of BD at age 24, with anterior uveitis and recurrent oral ulcers. | **- Neurological:** At age 32 years, dysarthria and difficulties in planning and executing activities.  **- Psychiatric:** At 32 years old, a diagnosis of undifferentiated schizophrenia was made (impoverished speech, affective flattening, apathy, neglect of self-care, social isolation, auditory hallucinations, behavioural disorganisation, and delusions of persecution). | **- Brain MRI:** Mild hyperintensities in the bilateral capsulostriate regions and left midbrain.  **- EEG:** No alterations. | **- General:** Steroids, cyclophosphamide, and methotrexate. The patient improved with azathioprine 100/150 mg every other day.  **- Psychiatric:** Haloperidol 6 mg/day, risperidone 6–8 mg/day, and olanzapine 20 mg/day without improvement. Ziprasidone 160 mg/day and aripiprazole 30 mg/day resulted in slight improvement. Finally, clozapine 300–400 mg/day was used with sustained clinical improvement despite mild sialorrhea. |
| De Vries & Van Vliet (2001) | Netherlands. | - ♂, 27 years old, originally from Morocco.  - No relevant clinical history. | **- Symptoms:** Onset years earlier, with recurrent oral ulcers, genital pustules, and uveitis. Diagnosed with BD at age 25.  **- Laboratory analytics:** Elevated ESR.  **- CSF:** Leukocytosis. | **- Neurological:** From the age of 25, drowsiness, Babinski (+) in the right foot, and cognitive impairment.  **- Psychiatric:** From age 25, mutism, strange behaviours, neglect of self-care, auditory and visual hallucinations. | **- Cranial MRI:** Dilated ventricles, hippocampal and temporal atrophy, alterations in white matter in the bilateral internal capsule.  **- EEG:** Slow theta and delta activity in the temporal region. | **- General:** Prednisone and cyclosporine.  **- Psychiatric:** Risperidone 4 mg/day for one month, without improvement. The cognitive decline was progressive. |
| Deniz et al. (2009) | Türkiye | - ♀, 18 years old (nationality not indicated).  - No relevant clinical history. | **- Symptoms:** Onset and diagnosis of BD at age 18 with orogenital ulcers along with neuropsychiatric symptoms.  **- Genital ulcer biopsy:** Lymphocytic vasculitis.  **- Pathergy test:** (+).  **- Laboratory analytics:** Normal.  **- CSF:** Normal. | **- Neurological:** At 18 years old, dysarthria, right hemiparesis, and right central facial paralysis.  **- Psychiatric:** At 18 years old, diagnosed with psychotic disorder (behavioural disturbances, visual and auditory hallucinations, social withdrawal, behavioural disorganisation, insomnia, irritability and poor insight). | **- Cranial CT scan:** normal.  **- Brain MRI:** hypointensities in the brainstem, right and left amygdala, right hippocampal gyrus, and posterior limb of the internal capsule. | **- General:** intravenous methylprednisolone 100 mg/day for 5 days and oral prednisone 60 mg/day, with improvement of neurological symptoms.  **- Psychiatric:** risperidone 2 mg/day, with slight improvement. |
| Dolapoglu & Kahya (2023) | Türkiye | - ♂, 32 years old, originally from Türkiye.  - No relevant clinical history. | **- Symptoms:** Onset and diagnosis of BD in 2017 with recurrent orogenital ulcers, arthralgia, thrombophlebitis, and uveitis. | **- Psychiatric:** At age 32 years, strange behaviours, soliloquies, auditory and visual hallucinations, neglect of self-care, insomnia and poor insight. | **- Brain MRI:** No alterations. | **- General:** Poor prior compliance with azathioprine, colchicine, enoxaparin, methylprednisolone, pantoprazole, and NSAIDs.  **- Psychiatric:** Sertraline 100 mg/day and aripiprazole 10 mg/day, with remission of symptoms. |
| Erdogan et al (2019) | Türkiye | - ♂, 38 years old (nationality not indicated).  - No relevant clinical history. | **- Symptoms:** Onset and diagnosis of BD at age 26 with recurrent oral ulcers, arthralgia, and bilateral blurred vision. | **- Neurological:** limbic encephalitis at age 36 and diagnosis of NBD.  **- Psychiatric:** At age 38 years, delusions, self-harm, jealousy, aggressiveness, impaired immediate memory, hypothymia, inappropriate affect, suicidal thoughts, and hallucinations. | **- Brain MRI:** No alterations.  **- EEG:** No alterations. | **- General:** Ciclosporin, azathioprine, lacosamide 300 mg/day, mycophenolate 2,000 mg/day.  **- Psychiatric:** Olanzapine 10–20 mg/day, with improvement of psychotic symptoms, boosted with haloperidol during the first 5 days. |
| Goolamali et al. (1976) | United Kingdom | - ♂, 26 years old (nationality not indicated).  - Recurrent respiratory infections, lymphadenopathy, and hepatosplenomegaly since childhood.  - Neurotic traits from adolescence and later, an established schizoid personality. | **- Symptoms:** Onset at age 2 with oral ulcers. Subsequently, genital ulcers, inflammatory eye lesions, and necrotic lesions developing at venipuncture sites.  **- CSF:** Normal.  **- Laboratory tests:** Slight increase in alpha 2 globulin and ESR.  **- Scrotal ulcer biopsy:** Chronic inflammation, necrosis, and acanthosis. | **- Psychiatric:** At age 26 years, diagnosed with schizophrenia with affective symptoms (insomnia, self-references, perplexity, crying, hypothymia, delusions, and hallucinations). | **- EEG:** Low amplitude beta tracing. | **- General:** Prednisone up to 60 mg/day with improvement in the number of exacerbations.  **- Psychiatric:** Electroconvulsive therapy, phenothiazines and antidepressants, with progressive improvement. |
| Haouala (2019) | Tunisia | - ♂, 44 years old (nationality not indicated).  - No relevant clinical history. | **- Symptoms:** Onset and diagnosis of BD at age 44 with recurrent oral ulcers and vascular involvement.  **- Pathergy test:** (+).  **-** **HLA B51:** (+) | **- Neurological:** At age 44 years, headache, left hemiparesis, and tetraparesis.  **- Psychiatric:** At age 44 years, anxious-depressive clinical presentation and visual hallucinations. At age 46 years, he was diagnosed with bipolar disorder type 1 after a manic episode. | - Not specified. | **- General:** Methylprednisolone in pulses, high-dose prednisone, and azathioprine.  **- Psychiatric:** Escitalopram 10 mg/day and risperidone 2 mg/day. Then amitriptyline 50 mg/day which induced a manic episode, treated with sodium valproate 1,500 mg/day. He had relapses due to poor compliance. |
| Hariri et al. (2010) | Türkiye | - ♀, 48 years old (nationality not indicated).  - No relevant clinical history. | **- Symptoms:** Onset and diagnosis of BD at age 32 with orogenital ulcers, arthralgia, and a corneal epithelium defect.  **- HLA-B5 and B51:** (+). | **- Psychiatric:** At age 41 years, depressive episode. She then experienced depressive, manic, and mixed episodes with rapid cycling and various hospitalisations. | - Not mentioned. | **- General:** Colchicine for 16 years.  **- Psychiatric:** Refractory treatment from age 42 to 48 years, unspecified. At age 48, valproate 1,000–1,500 mg/day, clonazepam 2.5 mg/day, and olanzapine 5 mg/day (discontinued due to metabolic syndrome) were used, the latter was replaced by aripiprazole 15 mg with mood stabilisation. After relapse, aripiprazole was increased to 30 mg/day and lithium carbonate 900 mg/day was added. |
| Hasbek et al. (2012) | Türkiye | - ♂, 41 years old (nationality not indicated).  - No relevant clinical history. | **- Symptoms:** Age of onset not specified but started with the presentation of orogenital ulcers and blurred vision. Diagnosis of BD one year later. | **- Neurological:** Two years after onset, difficulty swallowing, urinary incontinence, and poor balance. At age 41, the patient suffered right central facial paralysis and hyperreflexia.  **- Psychiatric:** At 40 years old, hypersexuality, disinhibition, and insomnia. At age 41, he experienced auditory hallucinations and was diagnosed with a manic episode due to a medical condition. | **- Brain MRI:** Multiple confluent lesions in the cerebral white matter and brainstem. | **- General:** Cyclosporine and colchicine. Then corticosteroids and azathioprine. Limited response in terms of the neurological symptoms.  **- Psychiatric:** Initially given zuclopentixol. Subsequently, quetiapine and valproic acid were used, with a good response in terms of the psychiatric symptoms. |
| Karroumi et al. (2024) | Morocco | - ♂, 33 years old (nationality not indicated).  - No relevant clinical history. | **- Symptoms:** Onset and diagnosis at age 26 with orogenital ulcers and uveitis. | **- Psychiatric:** From the age of 33, the patient presented persecutory and megalomaniacal delusions, disorganised language, tangential thinking, neologisms, agitation, and hostility. | **- Brain MRI:** Hypoperfusion in the left lateral sinus and superior sagittal sinus.  **- EEG:** No abnormalities. | **- General:** Azathioprine 50 mg/day.  **- Psychiatric:** Risperidone 4 mg/day and lorazepam 5 mg/day with partial improvement of the psychiatric symptoms at 6 months, persistence of negative clinical features (emotional indifference, social withdrawal, and pragmatism). |
| Koçer et al. (2007) | Türkiye | - ♂, 40 years (nationality not indicated).  - No relevant clinical history. | **- Symptoms:** Onset and diagnosis at age 40 with orogenital ulcers and uveitis.  **- Pathergy test**: (+). | **- Neurological:** From the age of 40 the patient presented headache, ataxia on the right side of the body, and loss of speech fluency. Cognitive impairment (memory, attention, and learning) and dyscalculia. Diagnosed with dementia.  **- Psychiatric:** From the age of 40, the patient experienced insomnia, disinhibition, and excessive spending. Diagnosed with depression. | **- Brain MRI**: cerebellar and brainstem atrophy.  **- Evoked potentials:** No alterations.  **- EEG:** No abnormalities. | **- General:** Prednisone 1 mg/kg/day and colchicine 1.5 mg/day, with partial improvement but difficulties in learning and temporal orientation persisted. Memantine was added, with improvement in cognitive impairment at 2 months.  **- Psychiatric:** Sertraline 100 mg/day. Partial improvement at 6 months. |
| Kurikawa et al. (2004) | Japan | - ♀ 67 years old (nationality not indicated).  - Admission to psychiatry for headache and depression. | **- Symptoms:** Onset and diagnosis at age 45 with Behçet’s intestinal disease and a diagnosis of BD. The patient presented with stomatitis and recurrent genital ulcers.  **- HLA B51:** (+).  **- CSF:** 2 cells/mm³. Proteins: 55 mg/dL. IgG/albumin ratio: 0.776.  **- Laboratory analytics**: elevated IgG: 2,701 mg/dL. ANA: 1/640. | **- Neurological:** From the age of 66, she developed chorea in the lower limbs. Bilateral hyperreflexia, Chaddock sign (+), headache. Cognitive impairment (Mini-Mental State Examination: 23)  **- Psychiatric:** From the age of 66 she presented disturbances in attention, delusions of persecution, hallucinations, depression, social isolation, and a subsequent suicide attempt. | **- Brain MRI:** multiple basal ganglia and white matter foci (hypointense on T1 and hyperintense on T2, ring enhancement on FLAIR).  **- MR angiography:** No abnormalities.  **- EEG:** diffuse slowing. | **- General:** prednisolone 60 mg/day. Initial improvement of chorea, with subsequent worsening.  **- Psychiatric:** maprotiline, zopiclone, diazepam, and etizolam. Partial initial improvement of psychotic symptoms, but subsequent worsening. |
| Maner et al. (2011) | Türkiye | - ♂, 43 years old (nationality not indicated).  - No relevant clinical history. | **- Symptoms:** Onset at age 23 with recurrent uveitis, oral ulcers, painful genital ulcers, and erythema nodosum.  **- HLA-B5:** (+) | **- Neurological:** Right hemiparesis due to stroke at age 37.  **- Psychiatric:** from the age of 37 he presented manic episodes with euphoria, psychomotor agitation, verbosity, insomnia, excessive spending, and megalomaniacal delusions. These symptoms intensified during uveitis flare-ups. Diagnosis of Bipolar Disorder type 1. | - Not mentioned. | **- General:** Azathioprine 150 mg/day (for 13 years). Prednisone 100 mg/day for one week during uveitis attacks.  **- Psychiatric:** Not specified. |
| Mirone et al. (1998) | Italy | - ♀, 20 years old (nationality not indicated).  - No relevant clinical history. | **- Symptoms:** The age of onset was not specified, but it began with presentation of recurrent oral ulcers, papules, and skin pustules.  **- Pathergy test:** (+) | **- Neurological:** Two years after the general symptoms, the patient presented recurrent headaches. Alterations in memory and concentration.  **- Psychiatric:** Auditory and visual hallucinations with delusional ideation and affective disorders. | **- Brain MRI:** No abnormalities.  **- SPECT:** Slight decrease in perfusion in the left frontal lobe.  **- EEG:** No abnormalities. | **- General:** Low doses of corticosteroids during flare-ups. Cyclosporine A (5 mg/kg per day), without improvement of the headaches.  **- Psychiatric:** Not specified. |
| Moreira et al. (2012) | Portugal | - ♀, 46 years old (nationality not indicated).  - No relevant clinical history. | **- Symptoms:** Onset at age 30 with asymmetric polyarthritis, erythema nodosum, orogenital ulcers, and uveitis, with a diagnosis of BD 2 years after onset. | **- Psychiatric:** Psychotic episode at age 32, followed 13 years later by a manic episode with disinhibition, insomnia, verbosity, delusions, and obsessive behaviour, resulting in a diagnosis of bipolar disorder. | **- Brain MRI** (45 years): Multiple hyperintense foci (T2, DTI, and FLAIR) in the subcortical white matter, compatible with an inflammatory process.  **- EEG:** No abnormalities. | **- General:** Steroids. Improvement of uveitis.  **- Psychiatric:** Initially olanzapine, subsequently adding valproate and sertraline with subsequent clinical stability. |
| Nkam & Cottereau  (2006) | France | - ♀, 31 years old, originally from Haiti.  - No relevant clinical history. | **- Symptoms:** No age of onset was indicated, but she began presenting oral ulcers, and later, genital ulcers, with a diagnosis of BD 1 year after onset. | **- Neurological:** Headache.  **- Psychiatric:** Onset at age 31. Psychotic disorder with delusions of persecution, megalomania, somatic delusions, and delusions of affiliation were described.  Exalted mood, agitation and hetero-aggressiveness, which over time turned into a depressive mood with negative symptoms (apathy, social isolation, etc.). | **- Brain MRI:** Absence of flow in the left lateral sinus, reduced flow in the superior sagittal sinus.  **- EEG:** No alterations. | **- General:** High-dose corticosteroids, colchicine, cyclophosphamide bolus. Low molecular weight heparin. Physical improvement in weeks. As maintenance treatment: colchicine 2 mg/day, prednisone 10 mg/day, azathioprine 100 mg/day.  **- Psychiatric:** Initially haloperidol (later discontinued) and clonazepam, with a good response. As maintenance:  Risperidone 2 mg/day, alprazolam 0.75 mg/day, zolpidem 10 mg/day, with partial remission of psychiatric symptoms but progression to residual symptoms with negative clinical presentation. |
| Odemir et al. (2004) | Türkiye | - ♂, 29 years old (nationality not indicated).  - No relevant clinical history. | **- Symptoms:** Onset at age 24 with orogenital ulcers and subsequent thrombophlebitis, with a diagnosis of BD 5 years after onset.  **- Pathergy test (+)** | **- Neurological:** Headache.  **- Psychiatric:** Onset around age 24, along with general symptoms. Depression with social withdrawal, anhedonia, and poor concentration was described. Followed by psychosis with delusions of persecution, megalomania and self-referentiality, thought theft phenomenon, Capgras syndrome, and auditory and visual hallucinations. | **- Brain MRI:** Subcortical changes in the white matter and meningeal structures.  **- EEG:** Brain dysfunction in the frontocentral areas. | **- General:** Initially warfarin, aspirin, and colchicine. Following the diagnosis of NBD, intravenous immunoglobulin was added.  **- Psychiatric:** Mianserin, risperidone (6 mg/day), biperiden (6 mg/day). Remission of psychotic symptoms and good social and family reintegration. |
| Ogawa et al. (1976) | Japan | Case 2 | | | | |
|  |  | - ♀, 42 years old (nationality not indicated).  - No relevant clinical history. | **- Symptoms:** The age of onset was not specified, but the patient first presented with oral ulcers, followed by genital ulcers and fever.  **- ESR:** Elevated **- CRP:** Elevated  **- CSF:** Increased protein. | **- Neurological:** Dysarthria, tremor, generalised spasticity, hyperreflexia.  **- Psychiatric:** Emotional instability, self-referential delusions. | **- EEG:** Diffuse alpha wave activity. | **- General:** Initially corticosteroids, later adding 6-mercaptopurine (6-MP) after relapse, with remission of cerebellar and sensory symptoms, but persistence of spasticity. |
|  |  | Case 3. | | | | |
|  |  | - ♂, 32 years old (nationality not indicated).  - No relevant clinical history. | **- Symptoms:** The age of onset was not specified, but he began presenting oral and scrotal ulcers and iritis, with a diagnosis of BD 1 year after onset.  **- ESR:** Elevated **- CRP:** Elevated  **- CSF:** Increased protein. | **- Neurological:** Headache, Weber’s syndrome, cognitive impairment, spastic paralysis of the limbs, and subsequently cerebellar symptoms (nystagmus, dysarthria, ataxia in the left limbs).  **- Psychiatric:** Personality changes with disinhibited and antisocial behaviours, aggressiveness, and emotional lability. | **- EEG:** Diffuse alpha wave activity. | **- General:** Corticosteroids and 6-mercaptopurine (6-MP), with remission of cerebellar symptoms and improvement in psychiatric and motor symptoms but persistence of left hemiparesis and mild memory impairment. |
| Orsucci (1996) | Italy | - ♂, 30 years (nationality not indicated).  - Father diagnosed with depression, died by suicide. | **- Symptoms:** No age of onset was indicated, but he began presenting with recurrent purpura and orogenital ulcers, with a diagnosis of BD after 6 months.  **- Immunological analyses:** Immune complexes, anti-endothelium, and anti-DNA autoantibodies.  **- HLA-B5:** (+). | **- Psychiatric:** Hypomania/mania with verbosity. Later, depression. | - Not mentioned. | **- General:** High-dose corticosteroids, with poor response. Colchicine and levamisole were subsequently used, with a mild response.  **- Psychiatric:** Psychotherapy, without effective psychiatric pharmacotherapy. Improvement after lifestyle changes. |
| Patel et al. (2013) | United States | - ♂, 17 years old, African American.  - No relevant clinical history. | **- Symptoms:** Onset and diagnosis of BD at age 17, presenting with a genital ulcer, episcleritis, and venous sinus thrombosis, in addition to neuropsychiatric symptoms.  **- ESR:** 30 mm/h  **- CRP:** 56 mg/L  **- CSF:** normal.  **- Pathergy test:** (−). | **- Neurological:** Headache with photophobia, generalised tonic-clonic seizures, and cognitive impairment.  **- Psychiatric:** Onset at age 17. Aggression, extreme mood swings, messianic delusions, auditory and visual hallucinations, compulsive hand washing, verbosity and tangential speech, and insomnia were described. | **- Cranial CT scan:** No alterations.  **- EEG:** No alterations.  **- Brain MRI:** Dural sinus thrombosis, grey and white matter lesions in the frontal, medio-occipital, and posterior left parietal lobes.  **- Cerebral MRI angiography:** No alterations. | **- General:** Anticoagulants and corticosteroids, plus lamotrigine and Dilantin for seizures. For maintenance, anticoagulants, corticosteroids, monthly infliximab and lamotrigine. Improvement of various symptoms.  **- Psychiatric:** Haloperidol initially, withdrawn after 5 months following improvement of the psychotic symptoms. |
| Shen et al. (2023) | China | - ♀, 43 years old (nationality not indicated).  - Sjögren’s syndrome, asthma, and type II diabetes. | **- Symptoms:** Onset and diagnosis of BD at age 28 with erythema nodosum, orogenital ulcers, and iridocyclitis.  **- HLA B51:** (+).  **- Laboratory analytics:** albumin ↓, γ-globulin ↑, α1-microglobulin ↑, TNF-α ↑ | **- Neurological:** Paresthesias in the lower extremities, drowsiness.  **- Psychiatric:** Since the age of 38, she has had two major depressive episodes with low mood, anhedonia, and abulia. At age 43, she experienced a new depressive episode and, during treatment, had a hypomanic episode with hyperthymia, tachypsychia, insomnia, and auditory hallucinations. | **- Brain MRI:** No alterations.  **- EEG:** No alterations. | **- General:** Methylprednisolone 8 mg/day and cyclosporine 200 mg/day, with clinical physical, and psychiatric improvement.  **- Psychiatric:** Venlafaxine for the management of the depressive episode, resulting in a hypomanic switch treated with quetiapine 300–400 mg/day, lurasidone 60 mg/day, and lithium carbonate 600 mg/day. Finally, maintenance on lithium carbonate monotherapy, stable after 6 months. |
| Tas et al. (2018) | Türkiye | - ♂, 47 years old (nationality not indicated).  - No relevant clinical history. | **- Symptoms:** No age of onset was indicated, but he was diagnosed after presenting blurred vision, oral ulcers, and genital ulcers. | **- Psychiatric:** Onset at age 46. Auditory hallucinations and a secondary suicide attempt were described. In addition, he was aggressive, irritable, and introverted. | - Not mentioned. | **- Psychiatric:** Sertraline 50 mg/day and subsequently quetiapine 100 mg/day and risperidone 3 mg/day. Improvement of the symptoms of psychosis. |
| Tosto et al. (2013) | Italy | - ♂, 65 years old (nationality not indicated).  - No relevant clinical history. | **- Symptoms:** Onset at age 46 with recurrent oral ulcers. At age 48, BD was diagnosed after presenting with panuveitis, erythema nodosum, and polyarthritis. | **- Neurological:** From age 50, progressive cognitive decline (mild-moderate) over several years.  **- Psychiatric:** From age 50, depression, apathy, irritability, and anxiety. | **- Brain MRI:** Although initially normal, during follow-up a lesion was observed in the mid-pons, which coincided with the onset of cognitive decline. | **- General:** Corticosteroids and cyclophosphamide.  **- Psychiatric:** Sertraline 100 mg/day. Improvement in mood and cognitive performance (Mini-Mental State Examination = 28). |
| Uhl et al. (1985) | United States | - ♂, 28 years old, originally from Iraq.  - No relevant clinical history. | **- Symptoms:** Onset at age 23, with orogenital lesions and epididymitis. At age 28, he was diagnosed with BD after experiencing loss of visual acuity and arthralgia for a year and a half.  **- ESR:** 66 mm/h.  **- Leukocytes**: 15,900/µL; | **- Psychiatric:** Onset at age 28 with delusional ideation and depression, in addition to marked weight loss secondary to delusions. This leads to a diagnosis of delusional disorder of organic origin. | **- EEG:** No alterations. | **- General:** Prednisolone 80 mg/day and dexamethasone eye drops, in addition to chlorambucil, with progressive improvement of uveitis.  **- Psychiatric:** Haloperidol 10 mg/day, with little improvement. Desipramine 150 mg/day was added, with mood improvement, but persistence of delirium. |
| Van Ham et al. 2014. | Belgium | - ♂, 34 years old, originally from Morocco.  - No relevant clinical history. | **- Symptoms:** No onset is indicated, but at the age of 30, he already presented neurological symptoms. At age 34, he was diagnosed with BD after presenting with orogenital ulcers and an erythematous rash on his chest.  **- Pathergy test:** (+).  **- HLA B51:** (+).  **- Laboratory analytics:** No alterations. | **- Neurological:** He presented gait disturbances at age 30.  **- Psychiatric:** Onset at age 34. Disinhibition, psychomotor agitation, accelerated speech, and insomnia were mentioned. This allowed for a diagnosis of a manic episode. | **- Cranial CT scan:** Small hypodense lesion in the right frontal region (residual from a previous biopsy).  **- Brain MRI:** Right frontal perivascular gliosis; early demyelination in the mesencephalic cerebral peduncle.  **- SPECT:** Hypoperfusion in the left frontal and temporal lobes.  **- EEG:** Mild nonspecific cortical dysfunction. | **- General:** Colchicine 1.5 mg, methylprednisolone 1 mg/kg, and azathioprine 100 mg/day. Azathioprine 50 mg monotherapy as a maintenance treatment was subsequently discontinued. Improvement of the ulcers.  **- Psychiatric:** Pipamperone 3×20 mg and lormetazepam 2 mg/day with insufficient response, therefore clotiapine 80 mg was added. Due to a lack of improvement, the previous medications were withdrawn and olanzapine 10 mg and valproic acid 1,000 mg/day were introduced, with significant improvement. |
| Verim et al. (2006) | Türkiye | -♀, 19 years old (nationality not indicated).  - Brother diagnosed with BD.  - Aunt with a history of OCD and an uncle with schizophrenia. | **- Symptoms:** Onset at age 19, with recurrent oral ulcers, genital ulcers, papulopustular lesions, and erythema nodosum.  **- CSF:** No alterations. | **- Neurological:** Hyperreflexia, positive Babinski, dysarthria, ataxic gait.  **- Psychiatric:** Onset at age 19 along with general symptoms. Capgras syndrome and mystical delusions, visual and auditory hallucinations, anger, insomnia, hyporexia, anhedonia. | **- Cranial CT scan:** No alterations.  **- Brain MRI:** Left cortical atrophy, 1 cm hyperintense lesion in the right cerebellar hemisphere (gliosis).  **- EEG:** Right temporal slowing. | **- Psychiatric:** Sertraline initially (poor adherence). Then quetiapine 400–600 mg/day. Progressive clinical improvement with partial persistence of psychotic symptoms at discharge. Maintenance with quetiapine 400 mg/day on an outpatient basis. |

**Table S3.** Neuroimaging findings reported in NPBD according to psychiatric phenotype.

| **Psychiatric Phenotype** | **Frequency** | **Reported Symptoms** | **Affected Brain Areas** |
| --- | --- | --- | --- |
| Psychotic disorders | ~8–10 cases  (≈25–30%) | Persecutory or grandiose delusions, auditory/visual/tactile hallucinations, thought disorganization, behavioral agitation, negative symptoms | Basal ganglia, amygdala, hippocampal gyrus, internal capsule, capsulostriatal regions, midbrain, brainstem, frontal and temporal hypoperfusion |
| Depressive disorders | ~6–8 cases  (≈20–25%) | Sadness, anhedonia, abulia, social withdrawal, suicidal ideation or attempts | Basal ganglia, white matter lesions, cortical or cerebellar atrophy, ventricular dilation, frontal hypoperfusion |
| Bipolar disorder | ~10–12 cases  (≈30–35%) | Euphoria, disinhibition, psychomotor agitation, insomnia, excessive spending, increased energy | Pontine lesions, brainstem involvement, cerebral white matter lesions, cerebellar atrophy |
| Cognitive impairment | ~5–7 cases  (≈15–20%) | Memory impairment, attention deficits, learning difficulties, temporal disorientation | Cortical and cerebellar atrophy, ventricular dilation, hippocampal and temporal lobe involvement, white matter lesions |
| Personality or behavioral changes | ~3–4 cases  (≈10–12%) | Odd behavior, aggression, mutism, disorganized or antisocial behavior | Frontal cortex dysfunction, anterior cingulate gyrus lesions, frontotemporal alterations |
| Obsessive–compulsive disorder | ~1–2 cases (≈3–6%) | Contamination obsessions, compulsive behaviors, tic-like symptoms | Fronto-striatal circuits including basal ganglia and frontotemporal junction, caudate nucleus |
| Somatoform disorders | ~1 case (<5%) | Severe anxiety, somatic complaints, appetite loss | Anterior cingulate gyrus, internal capsule lesions, limbic system involvement |

**Figure S1.** Simplified therapeutic decision algorithm for Neuro-psycho Behçet’s disease.
